# Problematic Use of Alcohol and Online Gaming as Coping Strategies During the COVID-19 Pandemic: A Mini Review

**DOI:** 10.3389/fpsyt.2021.685964

**Published:** 2021-06-14

**Authors:** Shijie Xu, Minkyung Park, Ung Gu Kang, Jung-Seok Choi, Ja Wook Koo

**Affiliations:** ^1^Medical Research Center, Hainan Cancer Hospital, Haikou, China; ^2^Institute of Human Behavioral Medicine, Medical Research Center, Seoul National University, Seoul, South Korea; ^3^Biomedical Research Institute, Seoul National University Hospital, Seoul, South Korea; ^4^Department of Psychiatry, SMG-SNU Boramae Medical Center, Seoul, South Korea; ^5^Department of Psychiatry and Behavioral Science, Seoul National University College of Medicine, Seoul, South Korea; ^6^Emotion, Cognition and Behavior Research Group, Korea Brain Research Institute, Daegu, South Korea; ^7^Department of Brain and Cognitive Sciences, Daegu Gyeongbuk Institute of Science and Technology, Daegu, South Korea

**Keywords:** alcohol, online gaming, addiction, COVID-19, pandemic, coping

## Abstract

The COVID-19 (coronavirus disease 2019) pandemic has dramatically changed our daily lives and activities, including those originally intended to serve for leisure and pleasure. Drinking and online gaming became coping behaviors used to rescue ourselves from the stress and restricted lifestyle during the COVID-19 pandemic. However, frequent drinking and gaming can result in the pathological consequences of addiction. Those affected use the stimuli not to obtain pleasure, but rather to avoid the displeasure induced by stress and previous use, often unsuccessfully. This review aims to provide an overview of recent longitudinal cohort studies on alcohol and gaming use during the COVID-19 pandemic, as well as to analyze how the pandemic has affected alcohol and gaming use. There was a substantial risk of alcohol and online gaming overuse during the lockdown, which may depend on the pandemic's duration or overuse patterns. Previous studies have shown that increased alcohol consumption and online gaming are associated with heightened stress and anxiety levels caused by social isolation/quarantine. Over time, frequent or excessive alcohol consumption and gaming could lead to an increased risk of more serious mental health problems. Every effort should be made to mitigate mental health problems and ensure adequate adaptation to these exceptional circumstances. Therefore, it would be helpful to encourage physical activity, social interaction, and collaboration to facilitate psychological and physical health.

## Introduction

The coronavirus (COVID-19) pandemic has caused unprecedented disruptions in the 21st century. Since this pandemic outbreak in 2019, our lives have radically changed, with serious consequences in terms of health, economy, and psychosocial perspectives. In response to the problems posed by the pandemic, governments have taken some emergency measures, including lockdown, restricting traffic, and closing down guilds and shops, which resulted in widespread depression, anxiety, and other adverse psychological reactions (1–5). Isolated and quarantined people frequently turn to substances (e.g., alcohol consumption) or rewarded behaviors (e.g., online gaming) to cope with their negative feelings (5, 6). Previous studies have reported that the impact of disasters, such as traumatic events (i.e., terrorism as 9/11), epidemic outbreaks (i.e., severe acute respiratory syndrome [SARS] in 2003), economic crises (2008's Great Recession), have resulted in increased rates of addictive behaviors, including alcohol drinking and problematic Internet use (7–9).

The pandemic is stressful in multiple aspects, and this stress may trigger increased alcohol intake in susceptible individuals (10, 11), leading to harmful neuroadaptations that further exacerbate alcohol craving. The use of rewarded behaviors/actions (e.g., online gaming) as putative coping strategies can also increase considerably in crises like the COVID-19 pandemic, and can develop into habits that are difficult to break (12).

Thus, the possible adverse effects of the COVID-19 pandemic on substance use and addictive behavior need to be carefully monitored. However, longitudinal research about alcohol/game use before and during the COVID-19 pandemic remain too scarce to elucidate risk factors associated with alcohol-/gaming-related problems observed over time. This narrative review discusses how the lockdown and quarantine by the COVID-19 pandemic affects addictive behaviors and associates negative consequences, focusing on up-to-date longitudinal cohort studies. This investigation would serve as a preliminary step for preventive measures against the mental health problems posed by COVID-19.

## Problematic Alcohol Consumption During the COVID-19 Pandemic

Several studies conducted using self-report measures during the early period of the COVID-19 pandemic have reported increased alcohol consumption during the COVID-19 pandemic, as summarized in Table 1. In an online survey of 1,074 Chinese participants, people in Hubei province (initial epicenter of the COVID-19 pandemic) showed an increased risk of harmful (11.1 vs. 1.9%) and hazardous alcohol use (33.5 vs. 21.5%), compared to people in other provinces after the COVID-19 outbreak (13), as measured by the Alcohol Use Disorders Identification Test (AUDIT) (23). In an online survey of 6,416 Chinese participants, almost one in five ex-drinkers relapsed, and 1.6% initiated alcohol abuse (14). Consistently, another online survey of 1,491 participants in Australia found that 26.6% of the respondents reported an increase in their alcohol consumption, which was mainly associated with higher levels of stress, anxiety, and depression symptoms (15). Although difficult to generalize (24), the findings of these online surveys with self-selected samples suggest that pandemic-related restrictions can be associated with higher drinking behavior.

**Table 1:** Literature review of alcohol consumption behavior during the COVID-19 pandemic.

**References**	**Country**	**Year**	**Sample size**	**Main findings**
Ahmed et al. (13)	China	April 2020	1,074 (aged 14–68 years)	In China, Hubei province had significantly higher proportions of harmful drinking (11.1 vs. 1.9%), hazardous drinking (33.5 vs. 21.5%), and AUD (6.8 vs. 1.0%) compared to other provinces.
Sun et al. (14)	China	From March 24–31, 2020	6,416 (mean age 28.23 ± 9.23 years)	In China, participants increased only marginally during the COVID-19 pandemic from 31.13 to 32.7% for alcohol drinking. Some 32.1% of regular drinkers reported an increased amount of alcohol drinking, 18.7% of ex-drinkers relapsed, and 1.7% of non-drinkers initiated the use of alcohol.
Stanton et al. (15)	Australia	From April 9–19, 2020	1,491 (mean age 50.5 ± 14.9 years)	In Australia, 22.3% of the participants reported consuming alcohol on ≤ 4 occasions per week. Of these, 26.6% reported increased alcohol consumption since the onset of the COVID-19 pandemic, which was associated with higher depression, anxiety, and stress.
Jackson et al. (16)	UK	Between April 2019–February 2020 and April 2020	20,558 (Adults)	In England, the prevalence of high-risk drinking (scores ≥5 as high-risk drinkers in AUDIT-C) increased during the COVID-19 lockdown compared to before the lockdown (25.1 vs. 38.3%).
Daly & Robinson (17)	UK	Between 2016–2018 and May 2020	3,358 (middle-aged)	In the UK, high-risk drinkers (scores ≥5 in AUDIT-PC) before the pandemic increased from 19.4 to 24.6% during the pandemic. The prevalence of drinking ≥4 times per week doubled from 12.5 to 26% between 2016–2018 and May 2020, respectively.
Niedzwiedz et al. (18)	UK	Between 2015 and 2020	9,748 (18 years of age or older)	In the UK Household Longitudinal study, the prevalence of psychological distress increased from 19.4% in 2017–2019 to 30.6% during the pandemic period. Frequent (≥4 times a week) and binge drinking (≥6 drinks on one occasion) increased more among women.
Suffoletto et al. (19)	U.S.A.	April 1, 2020	50 (aged 18–25 years)	In Pittsburgh, an online survey was conducted over 6 weeks during the social isolation. Participants with any in-person contact decreased alcohol drinking from 44 to 29% in the 1st week of stay-at-home, and increased to 65% by week 6 of the stay-at-home. Young adults who drank alcohol reported more in-person contacts compared to non-drinking days.
Korean Addiction Forum (20)	Korea	From May 20–29, 2020	1,017 (Adults)	In Korea, 54.2% of the respondents reported decreased alcohol drinking after COVID-19, with only 7.5% indicating an increase. Participants who drank fewer than four glasses of alcohol increased from 45 to 52.9% after COVID-19, while people who drank more than test glasses reduced from 23.3 to 17%.
Weerakoon et al. (21)	U.S.A.	Between April 29–June 9, 2019 and May 28–Jun 16, 2020	1,540 (aged 30–59 years)	A nationally representative sample using the RAND Corporation American Life Panel (ALP) reported that the frequency of alcohol consumption showed an increase of 0.74 days during the pandemic, as compared to the baseline of 5.48 days in 2019. Specifically, increases in heavy drinking days (0.18 vs. 0.07 days) and alcohol-related problems (39 vs. 27%; as measured by the Short Inventory of Problems Scale) were more evident in women than in men.
Korean Society for Traumatic Stress Studies (22)	Korea	From March to May, September, and December, 2020	4,079 (aged 19–70 years)	A mental health survey by the Korean Ministry of Health and Welfare reported that the prevalence of frequent alcohol consumption increased steadily over time during the pandemic (8.35% in March, 11.57% in May, 10.68% in September, and 15.02% in December). Furthermore, the quantity of alcohol use increased with the duration of the pandemic (6.34% in March, 9.72% in May, 9.48% in September, and 12.33% in December).

More recent studies using cross-sectional analyses with longitudinal cohorts have corroborated the adverse effects of the COVID-19 pandemic on alcohol drinking in the general population (see summary in Table 1). Monthly cross-sectional surveys of a representative sample of adult population in the UK (20,558 participants aged 16 years and above) found that the prevalence of high-risk drinking (AUDIT score ≥5) increased from 25.1% before the lockdown (April 2019–February 2020) to 38.3% during the lockdown (April 2020) (16). Another UK study, using data from *the 1970 British Cohort Study* (3,358 participants), a prospective cohort study of 17,000 children born in Britain in 1970, showed increases in high-risk drinking from 19.4 to 24.6% (in primary care settings, AUDIT score ≥5) and frequent drinking from 12.5 to 26% (prevalence of drinking ≥4 times a week) among middle-aged adults between 2016–2018 and May 2020 (17).

These detrimental effects of the COVID-19 crisis and its associated lockdown restrictions on drinking patterns (e.g., high-risk drinking, binge drinking, frequent drinking, etc.) are likely to be more pronounced among women. A study in the US with 1,540 members of *the RAND Corporation American Life Panel* (ALP), a nationally representative, probability-sampled panel of 6,000 participants (aged 18 years and above) found that increased heavy drinking, defined as having more than five (man) or four (woman) alcoholic drinks within a couple of hours, was more pronounced among women than men in May–June 2020 compared to April–June 2019. Consistently, women were more likely than men to have increased alcohol-related problems as measured by the Short Inventory of Problems scale (25). Furthermore, analyses of data between 2015 and 2020 from the *UK Household Longitudinal Study*, a nationally representative longitudinal panel study based on a clustered-stratified probability sample of UK households (9,748 participants aged 18 years and above), revealed that the COVID-19 lockdown (April 2020) increased frequent (≥4 times a week) and binge drinking (≥6 drinks on one occasion) particularly among women (18). The gender difference in alcohol consumption can be associated with the lifestyle and stress management during the pandemic. Life stressors, such as quarantine and social isolation, which are associated with anxiety and depression (26, 27), lead to an unhealthy lifestyle such as unhealthy diet, high-risk drinking, etc. (26, 28). Reportedly, quarantine led to weight gain and an increased incidence of depression and stress among women more than men (29). Additionally, the aforementioned UK household analysis showed that psychological distress, as assessed by the General Health Questionnaire-12 after the 1st month of lockdown, together with adverse alcohol use increased significantly among women (18). These gender differences may be explained by biophysiological mechanisms that enhance women's vulnerability to negative emotions and stress responses (30). Consequently, women tend to experience higher alcohol-related health problems than men, especially when women are heavy drinkers (31, 32). This reflects international trends indicating that the prevalence of alcohol-related harms is escalating among women (33).

Besides social isolation, other environmental factors have also been associated with distress and alcohol use during the COVID-19 pandemic. For instance, boredom and monotony are a hotbed for numerous psychological challenges. Struk et al. (34) found that feeling bored is a motive behind rule-breaking behaviors that can affect compliance with social distancing policies. A preliminary study in the US showed that young adults' (aged 18–25 years) non-adherence to social distancing policies is associated with hazardous alcohol drinking (19). On the other hand, most people following strict restriction policies and lockdowns have suffered from the disruption of their daily routines. In the absence of external cues such as office hours, they have difficulties in discriminating the periods that should be dedicated for work, leisure, or household tasks. Their circadian rhythm gets disrupted, leading to negative affect and stress (35, 36), which can induce significant alcohol use and craving (37). Furthermore, alcohol works as a self-medication for depression (38). However, the positive affect induced by alcohol use does not last long, while the negative consequences become prominent, including various drinking-related problems and exacerbated depression (39–41). Following a daily routine, engaging in regular physical activity, and improving coping skills are some protective factors for drinking behavior (42).

In South Korea, a preliminary survey of 1,017 participants showed that a majority (61.9%) of pre-pandemic heavy/frequent drinkers (≥4 times a week) showed increased alcohol consumption during the outbreak, while a majority of former moderate (64.7%), light (70.9%), or non-drinkers (55.8%) (≤3 times a week) showed decreased alcohol consumption (20). Similarly, an online survey conducted by the Norwegian Directorate of Health in June–July 2020 with 1,328 respondents (aged 18 years and above) selected randomly from a national web panel reported that the upper 5–10% of heavy drinkers (≥~8.8 drinks a week) during the COVID pandemic increased their alcohol consumption, thereby increasing the prevalence of heavy drinkers (43). Additionally, a European online survey (40,064 participants aged 18 years and older) in 21 European countries showed an increase of harmful alcohol consumption (AUDIT-C score ≥8), but not in low harmful alcohol consumption (AUDIT-C score ≤7) (44). Alcohol cravings among heavy/frequent drinkers can be further exacerbated over time during the quarantine. A study in the US with 5,931 participants recruited via an online crowdsourcing platform (45) showed notable increase in harmful alcohol use (AUDIT-C score ≥8) among those under lockdown over the 6 months of the 2020 COVID-19 pandemic. However, it was essentially unchanged for those not under restrictions (46). Noticeably, an online survey of 1,982 participants in the US reported that the duration spent at home during the COVID-19 pandemic was associated with odds of binge drinking (21), defined as having more than five (man) or four (woman) drinks on one occasion (47). Similarly, a mental health survey by the Korean Ministry of Health and Welfare recently reported that the frequency of alcohol use by heavy/frequent drinkers increased over time during the lockdown (22). These preliminary data should be treated with caution; however, they suggest detrimental effects of prolonged quarantine on alcohol use. It is crucial to monitor potential long-term changes in drinking habits over time during the COVID-19 pandemic.

## Problematic Online Gaming Use During the COVID-19 Pandemic

Online gaming and Internet use are also frequently employed as putative coping strategies to reduce the adverse effects of prolonged isolation and loneliness (48–50). Adequate online gaming activities to reduce negative feelings and communicate with others can benefit an individual's mental health (51, 52). According to an Australian survey, only 2.1% of the 2,004 participants reported negative consequences of video games, while 77.2% of the participants indicated that video games had a positive mental health impact, and that games have the potential to promote mental health and provide opportunities to interact with friends through online platforms (53).

However, excessive and uncontrollable online gaming may lead to serious psychological and neurophysiological functions (54). Studies related to problematic Internet use and online gaming use during COVID-19 have been reported (51, 55, 56), which were summarized in Table 2. In China, one survey reported that 46.8% of the 6,416 participants [aged 28.2 ± 9.2 (mean ± SD) years] showed increased scores on the Internet Addiction Test (IAT) (14, 63). Of the participants, 4.3% showed severe internet dependence (80 ≤ IAT score ≤ 100), which is 23% of increase in the prevalence compared to the pre-pandemic period (October 2019). Another survey with 2,050 Chinese adolescents (aged 12.3 ± 4.7 years) during the COVID-19 showed that 2.7 and 33.4% of them were classified as addictive Internet users (IAT score ≥70) and problematic Internet users (40 ≤ IAT score ≤ 69), respectively. Notably, the IAT scores were significantly associated with depression and stress (52). Fernandes et al. (57) reported that 185 adolescents (aged 21.6 ± 2.6 years) from several countries (e.g., India, Malaysia, Mexico, and the UK) have shown increased Internet use and high scores of gaming addiction, which is contributed to loneliness and depression. Indonesia's nationwide web-based study with 4,734 adults (aged 31.8 ± 7.7 years) found that the prevalence of Internet addiction during the COVID-19 crisis was 14.4% in adults, higher than before the pandemic. This study also identified that duration of Internet use, psychopathologies, and decreased sleep quality were predictable factors of Internet addiction during self-quarantine, especially in individuals with confirmed/suspected COVID-19 cases within households (58). Most recent longitudinal study with 1,778 members *the Project of School Mental Health* in Southwest corroborated that videogame use and Internet Gaming Disorder (IGD) were significantly increased during the COVID-19 (April–May 2020) compared to before the pandemic (October–November 2019). Notably, the results from this study suggested that depressive and anxiety symptoms at pre-pandemic period positively predicted videogame use and IGD during the COVID-19 (55).

**Table 2:** Literature review of online gaming use during the COVID-19 pandemic.

**References**	**Country**	**Year**	**Sample size**	**Main findings**
Sun et al. (14)	China	From March 24 to 31, 2020	6,416 (mean age 28.23 ± 9.23 years)	In China, 46.8% of the respondents showed increased dependence on Internet use, and 16.6% had longer hours of Internet use during the COVID-19 pandemic. Some 4.3% reported severe Internet addiction, which was 23% higher than the prevalence rate of addiction (3.5%) found before the COVID-19 pandemic.
Korean Addiction Forum (20)	Korea	From May 20 to 29, 2020	1,017 (Adults)	In Korea, 24% of the respondents reported increased online gaming use after COVID-19, and 50.7% of the severely depressed group spent more time on smartphones.
Panno et al. (49)	Italy	From March 9 to May 4, 2020	1,519 (mean age 28.49 ± 10.89 years)	In Italy, a self-report survey showed that social media addiction and alcohol problems were positively correlated with COVID-19 during the lockdown.
Dong et al. (52)	China	From February 19 to March 15, 2020	2,050 (aged 6–18 years)	Among Chinese children and adolescents during the COVID-19 pandemic, 2.68 and 33.37% of the participants were classified as addicted and possibly addicted to the Internet. Internet use was mainly influenced by the COVID-19 epidemic, including frequency and duration of recreational Internet use, and the rate of stay-up use.
Ellis et al. (53)	66 different countries (Including the United States, United Kingdom, Canada)	From May 15 to 29, 2020	2,004 (aged 18–99 years)	The participants reduced their exercise time from an average 7.5 h per week to 6.5 h, and increased video game time from 16.38 h per week on average to 20.82 h during the COVID-19 period. Note that 77.2% of the participants reported that playing video games had been beneficial to their mental health.
Fernandes et al. (57)	Several countries (Including India, Malaysia, Philippines, Mexico, the UK)	–	185 (aged 16–25 years)	Adolescents increased their use of social media sites and streaming services during the pandemic. Regardless of country of residence, COVID related worries, compulsive Internet use, social media use and gaming addiction, predicted scores of escapism, depression, and loneliness.
Siste et al. (58)	Indonesia	From April 28 to June 1, 2020	4,734 (aged 21–40 years)	A prevalence of IA (14.4%) among Indonesian adults during COVID-19. Internet use increased by 52% compared to before the pandemic. Increased Internet duration, specific Internet motives, psychopathologies, and decreased sleeping quality were correlated to IA during the COVID-19.
Teng et al. (55)	China	Between October–November, 2019 and April–May, 2020	1,778 (children and adolescents)	A longitudinal study from the Southwest Chinese children and adolescents reported that children and adolescents increased videogame use during the COVID-19 pandemic (April–May, 2020) in comparison to the pre-pandemic period (October–November, 2019), but only adolescents increased IGD severity, as measured by Internet Gaming Disorder Scale-Short Form. Importantly, pre-pandemic depressive and anxiety symptoms predicted both videogame and IGD severity during the pandemic.
Servidio et al. (59)	Italy	–	454 (aged 18–25 years)	Fear of COVID-19 was associated with Internet addiction disorder, and fear of COVID-19 mediated the relationship between anxiety and Internet addiction disorder.
Fazeli et al. (60)	Iran	From May 22 to August 26, 2020	1,512 (aged 13–18 years)	Depression, anxiety, and stress serve as strong mediators in the association between Internet gaming disorder, insomnia, and quality of life among adolescents during the COVID-19 pandemic.
Islam et al. (61)	Bangladesh	From May to June, 2020	13,525 (aged 18–50) years	Problematic Internet use was associated with socio-demographic factors (young adults, a higher level of education, living with a nuclear family, engaging in less physical activities, playing online videogames, and social media).
Korea Creative Content Agency (62)	Korea	From May 27 to June 15, 2020	3,084 (aged 10–65 years)	In Korea, 70.5% of the participants responded that they have played games and the time spent on digital games has increased amid the COVID-19 situation, and 4.8 percentage-points increased from a similar survey conducted a year ago.

A number of studies have confirmed the association between behavioral disorders (i.e., IGD, Internet addiction, and gambling disorder) and psychological factors such as depression, anxiety, and stress (56, 64). An Italian study revealed that fear of COVID-19 was associated with Internet addiction disorder, and fear of COVID-19 mediated the relationship between anxiety and Internet addiction disorder (59). A Chinese study reported that the levels of depression and stress were associated with severity of Internet addiction measured by IAT scores in children and adolescents during COVID-19 (52). An Iranian study demonstrated that psychological factors such as depression, anxiety, and stress significantly mediated the association between Internet gaming disorder and outcomes of insomnia and quality of life among adolescents (60). In addition, a large-scale study in Bangladesh reported that problematic Internet use during COVID-19 was associated with socio-demographic factors (age, educational qualifications, marital status, socioeconomic status) and lifestyle factors (smoking status and sleeping hours) (61).

Additionally, a preliminary nationwide survey of 1,017 participants by the Korean Addiction Forum (KAF) in South Korea, which was conducted between May 20 and 29, 2020, reported that ~24.4% of the respondents said they spent more time on online games after the COVID-19 pandemic (compared to 16.3% who spent less time on online games) (20). Of the respondents, 44.3% said that their smartphone use also increased. They normally use smartphones for communication and social networking. Furthermore, a later online survey (May–June 2020), conducted on 3,084 people aged from 10 to 65 by the Korea Creative Content Agency, showed that 70.5% of the respondents had played digital games since June 2019, with a 4.8% increase compared to the previous survey conducted a year ago (62). In terms of platform, gaming on mobile devices was the most popular means of gaming, accounting for 91.1%, followed by PC games (59.1%), and video game consoles (20.8%).

## Discussion

Preliminary studies indicated that increased alcohol consumption as a coping strategy could be associated with social isolation, boredom, and lack of a routine. However, heavy/frequent drinking was not associated with coping motives, but it was associated with greater alcohol problems (65, 66), underscoring the importance of monitoring solitary drinkers as they may be more vulnerable to negative consequences.

Nonetheless, some limitations in the present study regarding alcohol consumption, alcohol-related problems, and Internet gaming disorders should be considered. A majority of the literature we reviewed may lack superior quality because a pandemic encourages a rapid rather than a meticulous spread of information. Many studies utilized non-standardized self-selected samples. It has been more than a year since the outbreak of the pandemic. During this period, numerous papers pertaining to the psychological and behavioral effects of the pandemic have been produced hurriedly. The academic society has had insufficient time to perform systematized and comprehensive researches. However, it is time to summarize these results to obtain a comprehensive view on this subject.

A further concern is that if some individuals may develop, increase, or relapse into unhealthy patterns of gaming to relieve pandemic-related stress, self-isolation restrictions may inhibit help-seeking, while also presenting barriers for those in treatment. For this reason, we encourage the exploration of online/telehealth options, including those that promote social connections (67). It may also be important to make recommendations about the types of videogames that may better facilitate psychological and physical health, including those that encourage physical activity, social interaction, and collaboration.

In response to the COVID-19 pandemic, experts in the field of addictive disorders worldwide have published consensus guidelines for preventing problematic Internet use (48). Although Internet and online gaming use can be an adaptive coping strategy to reduce pandemic-related psychological problems for the majority, it can pose a potential risk to vulnerable people who are at high risk of developing problematic usage patterns (68). Excessive engagement in online gaming and Internet activities may contribute to serious functional impairment and a higher risk of developing addictive disorders during prolonged quarantine periods. Therefore, it is essential to maintain a moderate and controlled level of these behaviors through a well-balanced daily routine, especially during the COVID-19 crisis (69). Several studies have pointed out that various factors, including high levels of stress, depression, anxiety, and fear of COVID-19, are associated with problematic online game use, and coping strategies for vulnerable individuals and distressed mental health professionals during the COVID-19 pandemic are necessary. Vulnerable populations require mental health care to minimize the potential negative consequences of the pandemic. Children and adolescents are vulnerable during the COVID-19 crisis. In particular, adolescents with psychiatric disorders are at higher risk of developing addictive disorders due to COVID-19-related problems and difficulties in adjusting to the pandemic. Therefore, government, educational and health care professionals, as well as parents should monitor and guide children and adolescents to stabilize their mood and reduce negative mental health effects of the COVID-19 crisis (70).

So far, most studies during COVID-19 indicate an increase in alcohol consumption and online gaming behavior. Further studies are needed to develop an approach for follow-up to better identify individual vulnerabilities and long-term impacts of the current pandemic on addictive behaviors.

## Author Contributions

All authors conceptualized the manuscript. SX, MP, and JK reviewed the literature, edited and critically reviewed the manuscript, and approved the final version. UK and J-SC edited and critically reviewed the manuscript, and approved the final version. All authors contributed to the article and approved the submitted version.

## Conflict of Interest

The authors declare that the research was conducted in the absence of any commercial or financial relationships that could be construed as a potential conflict of interest.
