# Corrigendum: Problematic Use of Alcohol and Online Gaming as Coping Strategies During the COVID-19 Pandemic: A Mini Review

**DOI:** 10.3389/fpsyt.2022.898218

**Published:** 2022-04-25

**Authors:** Shijie Xu, Minkyung Park, Ung Gu Kang, Jung-Seok Choi, Ja Wook Koo

**Affiliations:** ^1^Medical Research Center, Hainan Cancer Hospital, Haikou, China; ^2^Institute of Human Behavioral Medicine, Medical Research Center, Seoul National University, Seoul, South Korea; ^3^Biomedical Research Institute, Seoul National University Hospital, Seoul, South Korea; ^4^Department of Psychiatry, SMG-SNU Boramae Medical Center, Seoul, South Korea; ^5^Department of Psychiatry and Behavioral Science, Seoul National University College of Medicine, Seoul, South Korea; ^6^Emotion, Cognition and Behavior Research Group, Korea Brain Research Institute, Daegu, South Korea; ^7^Department of Brain and Cognitive Sciences, Daegu Gyeongbuk Institute of Science and Technology, Daegu, South Korea

**Keywords:** alcohol, online gaming, addiction, COVID-19, pandemic, coping

In the original article, there was an error. We cited data from an “Australian” survey, not an “Austrian” study. A correction has been made to section ‘Problematic Online Gaming Use During the Covid-19 Pandemic’, paragraph one:

“According to an Australian survey, only 2.1% of the 2,004 participants reported negative consequences of video games.”

In the original article, there was a mistake in Table 2 as published. There is a typo in a 5th row. The date is not May 19th, but May 29th. Also, in the same row, we clarify the main finding of reference 53 about a beneficial effect of videogame to mental health. The corrected table appears below.

**Table 2:** Literature review of online gaming use during the COVID-19 pandemic.

**Authors**	**Country**	**Year**	**Sample size**	**Main findings**
Sun et al. (14)	China	From March 24 to 31, 2020	6,416 (mean age 28.23 ± 9.23 years)	In China, 46.8% of the respondents showed increased dependence on Internet use, and 16.6% had longer hours of Internet use during the COVID-19 pandemic. Some 4.3% reported severe Internet addiction, which was 23% higher than the prevalence rate of addiction (3.5%) found before the COVID-19 pandemic.
Korean Addiction Forum (20)	Korea	From May 20 to 29, 2020	1,017 (Adults)	In Korea, 24% of the respondents reported increased online gaming use after COVID-19, and 50.7% of the severely depressed group spent more time on smartphones.
Panno et al. (49)	Italy	From March 9 to May 4, 2020	1,519 (mean age 28.49 ± 10.89 years)	In Italy, a self-report survey showed that social media addiction and alcohol problems were positively correlated with COVID-19 during the lockdown.
Dong et al. (52)	China	From February 19 to March 15, 2020	2,050 (aged 6–18 years)	Among Chinese children and adolescents during the COVID-19 pandemic, 2.68 and 33.37% of the participants were classified as addicted and possibly addicted to the Internet. Internet use was mainly influenced by the COVID-19 epidemic, including frequency and duration of recreational Internet use, and the rate of stay-up use.
Ellis et al. (53)	66 different countries (Including the United States, United Kingdom, Canada)	From May 15 to 29, 2020	2,004 (aged 18–99 years)	The participants reduced their exercise time from an average 7.5 h per week to 6.5 h, and increased video game time from 16.38 h per week on average to 20.82 h during the COVID-19 period. Note that 77.2% of the participants reported that playing video games had been beneficial to their mental health.
Fernandes et al. (57)	Several countries (Including India, Malaysia, Philippines, Mexico, the UK)	–	185 (aged 16–25 years)	Adolescents increased their use of social media sites and streaming services during the pandemic. Regardless of country of residence, COVID related worries, compulsive Internet use, social media use and gaming addiction, predicted scores of escapism, depression, and loneliness.
Siste et al. (58)	Indonesia	From April 28 to June 1, 2020	4,734 (aged 21–40 years)	A prevalence of IA (14.4%) among Indonesian adults during COVID-19. Internet use increased by 52% compared to before the pandemic. Increased Internet duration, specific Internet motives, psychopathologies, and decreased sleeping quality were correlated to IA during the COVID-19.
Teng et al. (55)	China	Between October–November, 2019 and April–May, 2020	1,778 (children and adolescents)	A longitudinal study from the Southwest Chinese children and adolescents reported that children and adolescents increased videogame use during the COVID-19 pandemic (April–May, 2020) in comparison to the pre-pandemic period (October–November, 2019), but only adolescents increased IGD severity, as measured by Internet Gaming Disorder Scale-Short Form. Importantly, pre-pandemic depressive and anxiety symptoms predicted both videogame and IGD severity during the pandemic.
Servidio et al. (59)	Italy	–	454 (aged 18–25 years)	Fear of COVID-19 was associated with Internet addiction disorder, and fear of COVID-19 mediated the relationship between anxiety and Internet addiction disorder.
Fazeli et al. (60)	Iran	From May 22 to August 26, 2020	1,512 (aged 13–18 years)	Depression, anxiety, and stress serve as strong mediators in the association between Internet gaming disorder, insomnia, and quality of life among adolescents during the COVID-19 pandemic
Islam et al. (61)	Bangladesh	From May to June, 2020	13,525 (aged 18–50) years	Problematic Internet use was associated with socio-demographic factors (young adults, a higher level of education, living with a nuclear family, engaging in less physical activities, playing online videogames, and social media).
Korea Creative Content Agency (62)	Korea	From May 27 to June 15, 2020	3,084 (aged 10–65 years)	In Korea, 70.5% of the participants responded that they have played games and the time spent on digital games has increased amid the COVID-19 situation, and 4.8 percentage-points increased from a similar survey conducted a year ago.

The authors apologize for these errors and state that they do not change the scientific conclusions of the article in any way. The original article has been updated.

## Publisher's Note

All claims expressed in this article are solely those of the authors and do not necessarily represent those of their affiliated organizations, or those of the publisher, the editors and the reviewers. Any product that may be evaluated in this article, or claim that may be made by its manufacturer, is not guaranteed or endorsed by the publisher.

